# Supplementary figures and images for: Impact of Cabozantinib Exposure on Proteinuria and Muscle Toxicity in Patients with Unresectable Hepatocellular Carcinoma
Source: Pharmaceuticals (Basel). 2022 Nov 25;15(12):1460. doi: 10.3390/ph15121460 (PMC9783864; doi:10.3390/ph15121460)

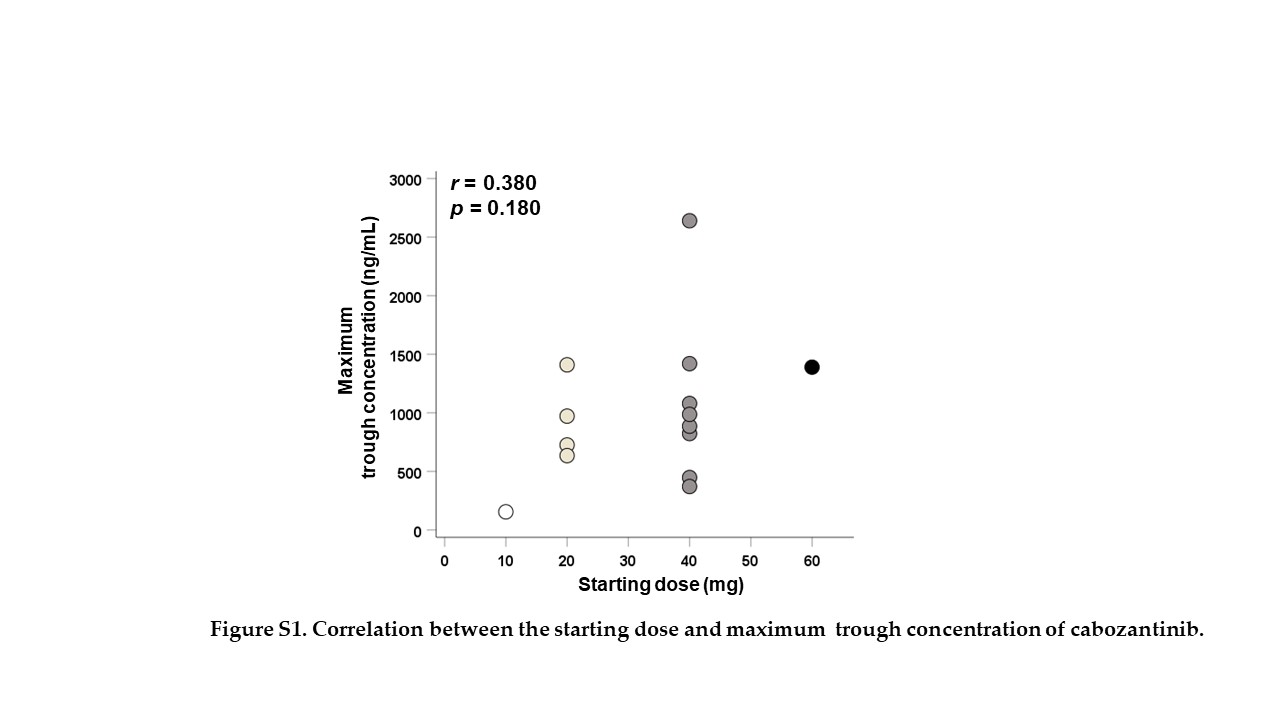

Supplement: Supplementary file 1 [file pharmaceuticals-15-01460-s001.zip › pharmaceuticals-2002652-supplementary.jpg]
